# A comparative finite element analysis of maxillary expansion with and without midpalatal suture viscoelasticity using a representative skeletal geometry

**DOI:** 10.1038/s41598-019-44959-w

**Published:** 2019-06-11

**Authors:** R. S. Fuhrer, D. L. Romanyk, J. P. Carey

**Affiliations:** 1 Department of Mechanical Engineering, Faculty of Engineering, University of Alberta, Edmonton, Alberta Canada; 2 School of Dentistry, Faculty of Medicine and Dentistry, University of Alberta, Edmonton, Alberta Canada; 3 Department of Mechanical Engineering, Faculty of Engineering, University of Alberta, Edmonton, Alberta Canada

**Keywords:** Biomedical engineering, Tissues

## Abstract

The goal of this investigation was to adapt and incorporate a nonlinear viscoelastic material model representative of the midpalatal suture’s viscoelastic nature into finite element analysis simulations of maxillary expansion treatment. Step-wise displacements were applied to a partial skull geometry to simulate treatment using an expansion screw appliance. Four simulation cases were considered for the midpalatal and intermaxillary sutures: 1. Neglecting suture tissue; 2. Linear elastic properties; 3. Viscoelastic properties; 4. A fused intermaxillary and viscoelastic midpalatal suture. Results from simulations indicated that removal of suture tissue and inclusion of viscoelastic properties resulted in the same maxillary displacement following 29 activations of 0.125 mm applied directly to the maxilla; however, assuming a fused intermaxillary suture significantly changed maxillary displacement patterns. Initial stress results within the suture complex were significantly influenced by the inclusion of suture viscoelasticity as compared to linear elastic properties. The presented study demonstrates successful incorporation of suture viscoelasticity into finite element analysis simulations of maxillary expansion treatment, and elucidates the appropriateness of various suture material property assumptions depending desired research outcomes.

## Introduction

**Figure 1:**
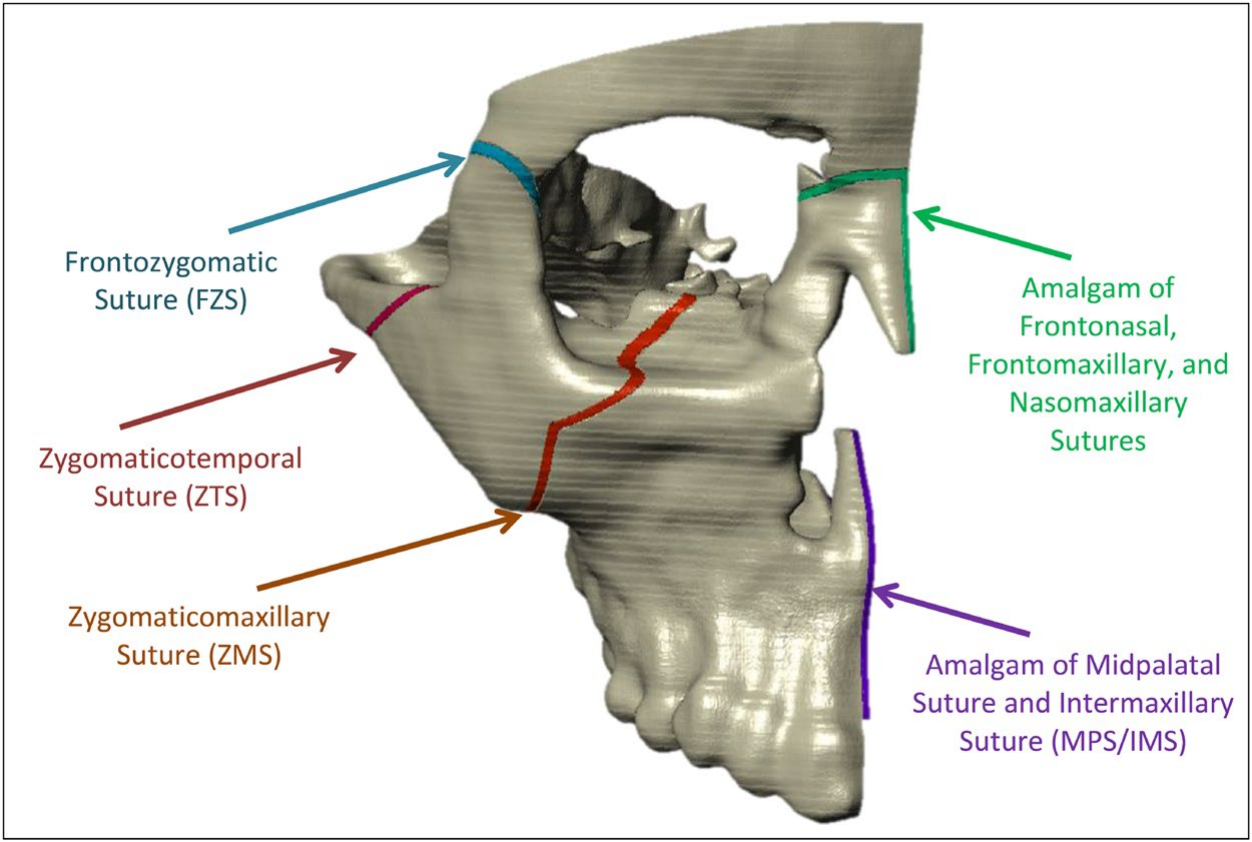
Partial skull geometry illustrating relevant cranial sutures and their approximate anatomical locations.

Maxillary expansion (ME) has been in use since 1860^1^ to mechanically widen the upper jaw, maxilla, of patients to aid in alleviating malocclusion of the dental arch^2^, nasal respiratory restrictions, and sleep apnea^3,4^. In terms of orthodontics, the primary focus of this study, commonly the purpose of ME treatment is to generate more space around the arch allowing the clinician to correct tooth misalignments. Widening the maxilla in turn expands the midpalatal suture (MPS) and intermaxillary suture (IMS) which are located at the midline of the maxilla as shown in Fig. 1. Structurally, the unfused MPS/IMS are connective tissue consisting primarily of collagen, extra-cellular matrix, and vasculature, and behave in a viscoelastic manner in response to externally applied forces^5,6^. ME treatment can be performed on a wide range of patient ages; however, the presented manuscript will consider the adolescent age range where the MPS/IMS structure has generally not ossified.

Finite Element Analysis (FEA) has been used previously to study the mechanics of ME treatment from several perspectives such as understanding stress distribution throughout the skull or the amount of maxillary widening that may be expected for a given simulated protocol^7^. One element missing from previous FEA studies surrounding ME treatment is that they neglected to incorporate the viscoelasticity of the MPS/IMS^7^. Suture material properties have been prescribed commonly either by (1) removing the structure completely^8,9^, (2) assigning linear^8,10^ or bilinear^9^ elastic properties, or (3) artificially reducing the accumulated stresses to zero^11,12^ – an assumption yet to be verified. Depending on the specific goals of the ME simulation, which are case dependent, these approaches may prove sufficient. As of yet, the effect of incorporating a representative model of MPS/IMS viscoelasticity in FEA simulations of ME, and the influence it may have on stress distributions within the MPS/IMS structure and the amount of predicted expansion, has yet to be explored. Investigating this research question would elucidate how MPS/IMS material model assignment influences ME treatment simulation and provide a better understanding of how to assign suture properties based on the specific study aims.

Romanyk *et al*. characterized the bulk material behavior of the midsagittal suture in New Zealand white rabbits using a 1D stress relaxation material model given as (Eq. (1))^13^:

1 $${\sigma }_{R}({\varepsilon }_{{0}_{R}},{t}_{w})=0.4894{(0.2880{\varepsilon }_{{0}_{R}}{t}_{w}^{-0.4912})}^{\frac{1}{0.4894}}$$

This model characterizes the relaxation stress, *σ*_*R*_, as a function of the initial suture strain, ${\varepsilon }_{{0}_{R}}$, and time, *t*_*w*_ (weeks). The model was determined using analytical methods to interrelate the nonlinear stress relaxation function in Eq. (1) to a creep strain function fit to the New Zealand white rabbit experimental creep strain data. Previous work using this model of suture stress relaxation predicted that MPS stress would decay rapidly to negligible values upon a step-increase of strain indicative of ME treatment, but was not tested within a representative geometry.

The presented study aimed to adapt the analytical 1-D stress relaxation model, developed by Romanyk *et al*. using an animal analog, in a FEA simulation using a representative cranial geometry. Results from simulations including MPS viscoelasticity will be compared to other commonly used methods to observe what effect this has on the mechanics of a simulated ME treatment. This research aims to better understand how choice of MPS/IMS material properties influences FEA simulation outcomes when considering ME treatment through step-wise increments in expansion (e.g. a hyrax screw appliance).

## Methods

### 1-D stress relaxation model adaption for use in finite element analysis

The stress relaxation model in Eq. (1) was adapted for implementation in the ANSYS Mechanical APDL (ANSYS® Academic Research, Release 14.5.7) FEA program. The first step in adaptation required a geometry correction factor, *γ*, to be derived. This factor was necessary as the development of Eq. (1) assumed a width larger than typical MPS values – a limitation of prior work^14^. The correction factor, *γ*, needed to account for a change in the initial suture width from the 9.72 mm dimension used by Romanyk *et al*.^14^ to 1.72 mm. This modification assumes the width of the original suture region used by Romanyk *et al*. was not fully comprised of suture tissue and contained 8 mm of bone which is significantly stiffer than suture tissue. The geometrically derived constant, *γ* = 0.176955, was determined as a ratio of the new assumed suture width, 1.72 mm, to the one assumed in previous work, 9.72 mm, to create the improved stress relaxation model (Eq. (2)).

2 $${\sigma }_{\gamma }({\varepsilon }_{{0}_{\gamma }},{t}_{w})=0.4894{(0.2880\gamma {\varepsilon }_{{0}_{\gamma }}{t}_{w}^{-0.4912})}^{\frac{1}{0.4894}}$$

The differences between the model presented in Eqs (1) and (2) are seen in the addition of the dimensionless factor, *γ*, and the substitution of the strain in the corrected suture width, ${\varepsilon }_{{0}_{\gamma }}$, for the strain in the originally assumed suture width, ${\varepsilon }_{{0}_{R}}$. Finally, *σ*_*γ*_ denotes the geometrically-corrected stress relaxation function. The stress relaxation model equation is only valid for *t* > 5 s as *σ*_*γ*_ → ∞ when *t*_*w*_ approaches zero.

The time dependency of Eq. (2) was approximated using a Generalized Maxwell viscoelastic model in order to utilize existing ANSYS material subroutines. The Maxwell Cauchy stress equation is given as (Eq. (3)):

3 $${\sigma }_{C}={\int }_{0}^{t}2G(t-\tau )\frac{de}{d\tau }d\tau \,+I{\int }_{0}^{t}2K(t-\tau )\frac{d{\rm{\Delta }}}{d\tau }d\tau \,$$

The variable *τ* in Eq. (3) is defined as the time at the end of the previous load sub-step, and *t* is the simulation time. *I* is the identity tensor and *σ*_*c*_ is the Cauchy stress. *G*(*t* − *τ*) and *K*(*t* − *τ*) are the shear and bulk moduli, respectively. Variables *e* and Δ signify the deviatoric and volumetric strains, respectively. A Prony expansion series was used in ANSYS to represent the time-dependent moduli terms of Eq. (3), specifically *G*( − *τ*) and *K*(*t* − *τ*), as shown in Eq. (4).

4 $$\begin{array}{cc}G({t}_{P})={G}_{0}[{\alpha }_{\infty }^{G}+{\sum }_{i=1}^{{n}_{G}}{\alpha }_{i}^{G}\exp (-\frac{{t}_{P}}{{\tau }_{i}^{G}})] & K({t}_{P})={K}_{0}[{\alpha }_{\infty }^{K}+{\sum }_{i=1}^{{n}_{K}}{\alpha }_{i}^{K}\exp (-\frac{{t}_{P}}{{\tau }_{i}^{K}})]\end{array}$$

In Eq. (4), *G*_0_ and *K*_0_ are the shear and bulk moduli values, respectively, at *t*_*p*_ = 0. The term (*t* − *τ*) from the Maxwell model was passed to the Prony series approximations (Eq. (4)) as the input variable, *t*_*P*_. The *α* terms, ${\alpha }_{i}^{G}\,$and ${\alpha }_{i}^{K}$, are the relative moduli. The *τ* terms, ${\tau }_{i}^{G}$ and ${\tau }_{i}^{K}$, are the relaxation time constants. The *n* variables, *n*_*G*_ and *n*_*K*_, signify the order of the Prony series expansion. The coefficients in Eq. (4) were determined utilizing the curve fitting functionality within ANSYS along with a stress-time data series generated using Eq. (2). The stress dataset had 10000 pairs of data points for the time range 5 s ≤ *t* ≤ 6 hrs. Two cases were considered where in one instance no time shift was applied to the Prony expansion series, and in the other the time value in the dataset was shifted by −4.99 seconds to account for the displacement ramp time. Results from both cases were compared to the expected results from the analytical solution in previous work to observe which provided a better representation. A 7-term Prony series expansion of Eq. (4) was utilized, resulting in the coefficients presented in Table 1.

**Table 1:** Calculated Prony Series Expansion Coefficients for Eq. (4) for Time Shifted and Non-Time Shifted Series Using a Stress-Time Data Set from Eq. (2) (Coefficients are as-reported when using the ANSYS curve fitting tool).

*Non-Time Shifted Series*				*Time Shifted Series*			
*t*_*d*_ = *t*_*c*_				*t*_*d*_ = *t*_*c*_ − *4.99 s*			
5 s <= *t*_*c*_ < 10000 s				5 s <= *t*_*c*_ < 10000 s			
*α*_1_	0.79591	*τ*_1_	5.000	*α*_1_	0.3927	*τ*_1_	2.8
*α*_2_	0.14287	*τ*_2_	20.379	*α*_2_	0.40143	*τ*_2_	10.376
*α*_3_	0.039795	*τ*_3_	62.859	*α*_3_	0.14725	*τ*_3_	39.696
*α*_4_	0.013401	*τ*_4_	173.230	*α*_4_	0.042999	*τ*_4_	149.81
*α*_5_	0.0050017	*τ*_5_	457.191	*α*_5_	0.011634	*τ*_5_	575.46
*α*_6_	0.0020479	*τ*_6_	1265.574	*α*_6_	0.0030511	*τ*_6_	2310.1
*α*_7_	0.00084392	*τ*_7_	4897.945	*α*_7_	0.0008232	*τ*_7_	114455
*β*	2.364188177			*β*	1.127029		
*t*_*int*_	5.6448 seconds			*t*_*int*_	5.6691 seconds		
*E*_0_	39.4864 MPa			*E*_0_	18.7425 MPa		

To utilize the Prony series expansion, Eq. (4), in ANSYS to simulate MPS stress relaxation, an initial Young’s Modulus, *E*_0_, is required. The Prony series forms an approximation of the analytical stress relaxation model, and as such they are not identical for values of time; however, the two models do intersect at several time values, which is be referred to as *t*_*int*_. This allows the relationship in Eq. (5) to be stated.

5 $${E}_{0}({\varepsilon }_{0})=\frac{{\sigma }_{Prony}({\varepsilon }_{0},{t}_{0})}{{\varepsilon }_{0}}=\frac{{\beta }_{{t}_{int}}{\sigma }_{Prony}({\varepsilon }_{0},{t}_{int})}{{\varepsilon }_{0}}=\frac{{\beta }_{{t}_{int}}{\sigma }_{\gamma }({\varepsilon }_{0},{t}_{int})}{{\varepsilon }_{0}}$$

where *σ*_*Prony*_ is defined as the stress relaxation approximated throughout the Prony series expansion, and ${\beta }_{{t}_{int}}$ is defined as the ratio of *σ*_*Prony*_(*t*_0_) and *σ*_*Prony*_(*t*_*int*_) through rearranging Eq. (5). *β* can thus be determined by substituting Eq. (4) into Eq. (5) and utilizing the fact that at *t*_*p*_ = 0 → *G(t*_*p*_) = *G*_0_, which results in Eq. (6):

6 $${\beta }^{G}({t}_{int})=\frac{1}{{\alpha }_{\infty }^{G}+{\sum }_{i=1}^{n}({\alpha }_{i}^{G}+{e}^{\frac{{t}_{int}}{{\tau }_{i}^{G}}})}$$

Finally, the initial Young’s Modulus, *E*_0_, value can be determined, as shown in Eq. (7). The initial strain value utilized was the average strain generated in the FEA model’s suture following the initial application of expansion displacement. The intercept time was found by determining the time of the first intercept of the Prony series expansion shear moduli function (Eq. (4)) and the shear moduli of the dataset used for curve fitting.

7 $${E}_{0}({\varepsilon }_{0},{t}_{int})=\frac{\beta ({t}_{int})\ast 0.4894{(0.2880\ast \gamma \ast {\varepsilon }_{0}\ast {t}_{int}^{-0.4912})}^{\frac{1}{0.4894}}}{{\varepsilon }_{0}}$$

**Figure 2:**
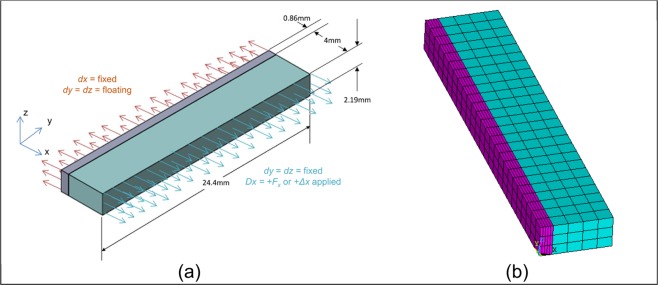
(**a**) Rectilinear Testing Geometry (RTG) model illustrating dimensions and boundary conditions and (**b**) the resulting mesh using 8-node brick elements (SOLID187). The simulated sagittal plane is located at the symmetric boundary conditions imposed on the suture elements.

A FEA model with a basic Rectilinear Test Geometry (RTG) was created based on the cross-sectional dimensions utilized by Romanyk *et al*.^14^ to verify that the adapted viscoelastic suture model worked as expected. The RTG is detailed in Fig. 2, and was developed utilizing symmetry across what would be the sagittal plane. 8-node brick elements, SOLID187 in ANSYS, were used to generate the mesh illustrated in Fig. 2. Simulations applied expansion displacements of 0.125 mm every 6 hours, with each application being ramped over the course of 5 seconds, for a total of 29 sequential activations. ANSYS static solution engine was chosen based on negligible difference in output results within trial simulations comparing it and the transient solution engine. The full-tangent Newton-Raphson solution method was used with a sparse-direct solver.

### Incorporation of adapted stress relaxation model in a partial skull geometry

**Figure 3:**
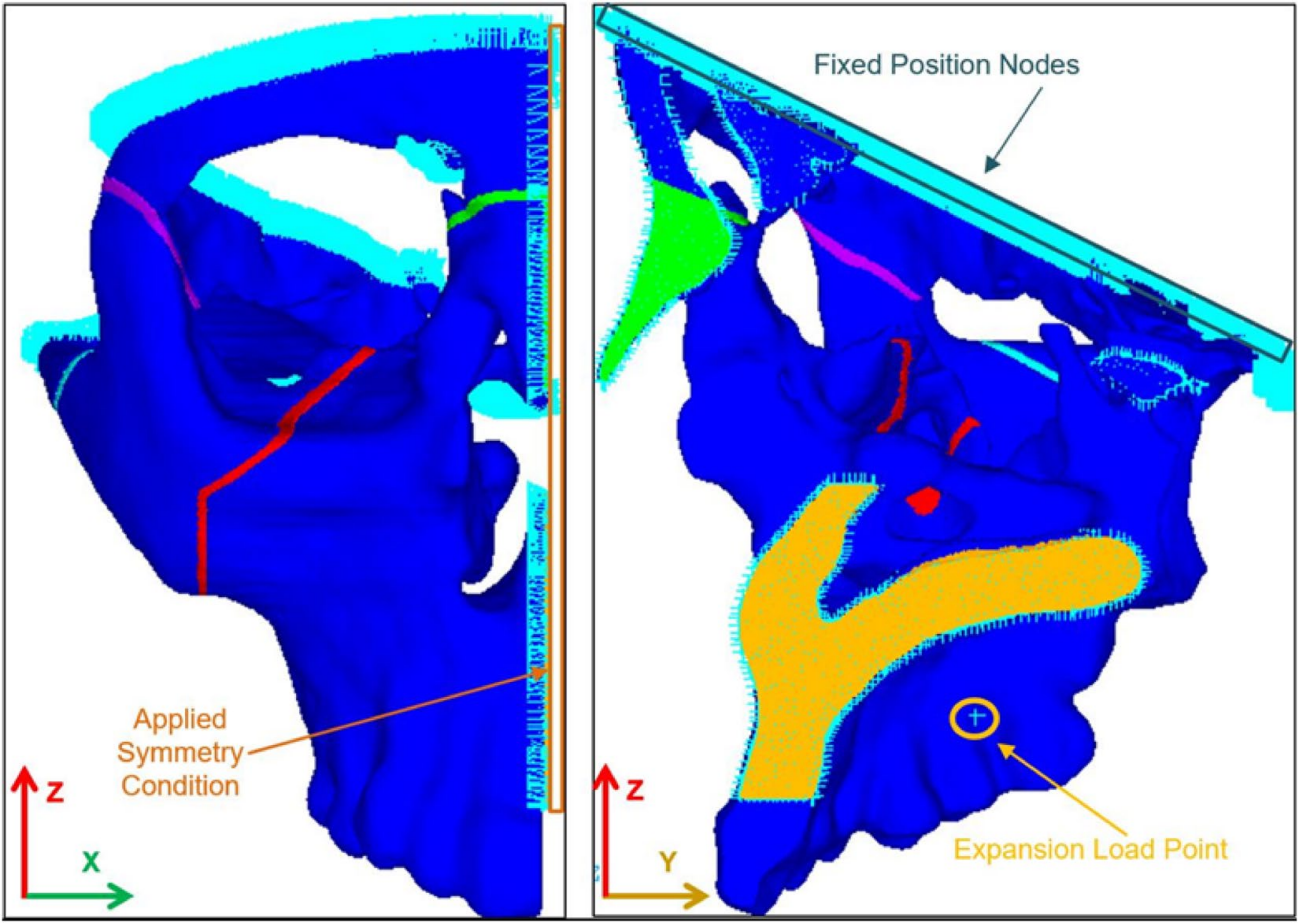
Partial skull geometry generated from pre-ME treatment CBCT scans illustrating fixed and symmetry boundary conditions, and showing MPS/IMS suture geometry for (**a**) anterior-posterior (**b**) medio-lateral views.

The adapted viscoelastic suture model was implemented in a partial skull geometry to explore the effect of various prescribed material conditions for the MPS/IMS structure on simulation results. Cone Beam Computed Tomography (CBCT) scans were used to create a pre-ME treatment skull geometry using the SimpleWare Scan IP + FE software package (Simpleware Ltd, Exeter, UK). All details regarding CBCT imaging methods are provided in previous work^2^. The scans were used under research ethics approval number PRO-00013379 from the University of Alberta Research Ethics Board, and patient informed consent was obtained. After rotating the scan dataset such that the sagittal plane would align with the global xy-plane, the geometry was semi-automatically masked from the CBCT images in Scan IP. Geometry refinement involved removing the mandible, superior regions of the skull, and smoothing the rough mask layers. The model was then segmented to separate and identify regions of bone and MPS/IMS suture followed by meshing of the partial skull geometry. The final FEA model, depicted in Fig. 3, was meshed with 4-node tetrahedral elements, SOLID185 in ANSYS, compatible with large non-linear deformations, and non-linear material models such as the Prony series expansion used in this study. An initial mesh of 210,880 elements was compared with one using 659,545 elements to investigate mesh density convergence. When required, the nonlinear solver utilized in this study was a Newton-Raphson method using the ANSYS default convergence criteria (i.e. having a residual vector less than 0.0005 for changes in forces and moments and 0.000001 for changes in element volume between load steps for convergence). Additionally, automatic time stepping was implemented.

Nodes on the superior and posterior cutting planes, shown in Fig. 3, were fixed in space to provide boundary conditions where the model boundaries would meet removed skull material. The fixed boundary condition was applied in this manner as it is far removed from the region of interest, provides the necessary fixed condition for solution, and removes unnecessary material from the simulation allowing for denser mesh in more critical areas. The sagittal cutting plane was fixed in the x-direction to impose a symmetry boundary condition. Additionally, a cluster of nodes were selected at the mid-root level between the second pre-molar and the first molar, and then connected to a single external node via 2-node bar elements with a stiffness three orders of magnitude larger than bone. The simulated ME activations were applied directly to the single external node, which then engaged the maxilla through the bar elements. By implementing displacement increments in this manner, it remedied local mesh nonlinearities and large elemental distortion that arose during preliminary methods where a uniform displacement was applied over all nodes in the loading region. Expansion displacements of 0.125 mm at each load step were applied in the x-direction, as defined in Fig. 3. A total of 29 load steps, representing simulated expansion activations, were applied with 12 hours between each load step. Activations were ramped over 5 seconds to simulate the time taken to activate the ME screw appliance.

Linear elastic properties of bone have been shown to be in the range of 10–20 GPa^15^. For the purposes of this study, the higher end linear elastic modulus of 20 GPa was utilized as an increased elastic modulus would be expected under rapid loading as simulated here (i.e. step increases in displacement). Four simulation cases using the partial skull model were considered in this study. Case 1 served as a baseline case, neglecting the MPS and IMS by removing the boundary conditions on their midsagittal plane nodes. Case 2 utilized compliant linear elastic properties of 1.27 MPa for the MPS and IMS, utilized by Romanyk *et al*.^14^ as an average of rabbit facial sutures determined by Radhakrishnan and Mao^16^. Case 3 employed the Prony series expansion of the nonlinear viscoelastic model (Table 1) for the MPS and IMS sutures, assuming the IMS was not fused. Case 4 replicated the viscoelasticity of the MPS, but assumed that the IMS had fused and was assigned properties of bone. In all aforementioned cases, all other cranial sutures were assumed fused. All materials were assumed isotropic and homogeneous. Mesh density convergence was studied using a single activation of Case 2 and comparing the average nodal first principal nodal stress using 27 locations across the MPS.

## Results and Discussion

### Verification of adapted stress relaxation model on rectilinear testing geometry

**Figure 4:**
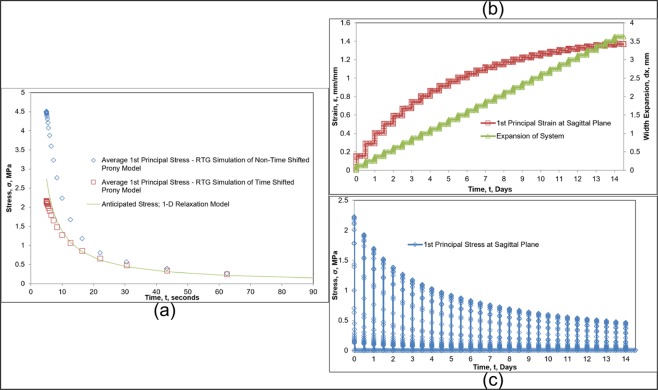
(**a**) Initial activation stress relaxation comparison between the theoretical 1-D model and time shifted and non-time shifted Prony series expansion; (**b**) Simulated maximum strain and suture expansion of the RTG using a time shifted Prony series approximation of suture viscoelasticity over 29 activations; (**c**) Maximum first principal stress at the suture sagittal plane over 29 activations.

Before incorporating any viscoelastic material properties in a partial skull model for ME treatment simulation, the RTG was used to ensure that the user-defined properties worked as expected on a simplified and well-understood geometry. The analytical model developed in previous work (Eq. (1)) was based on experimental data, and thus comparison of the FEA model to past results using the same formulation would verify the user-defined viscoelastic material model has been established correctly in ANSYS. Figure 4a compares the first simulated activation stress response of the FEA model against the expected stress profile as generated by the original Romanyk *et al*. 1-D model for an identical suture expansion. The peak stress at 5 seconds for the time shifted Prony expansion replicates that of the original validated analytical model. The difference in results is suggested to be a result of the 5 second ramped activation in the FEA simulation which is expected to cause a lower-magnitude initial stress than the 1-D model which assumed a step-increase. The non-time shifted series expansion resulted in a significantly larger initial stress prediction and did not follow the stress relaxation behavior as well as the time shifted expansion. This result enforces the necessity to incorporate a time shift in the series expansion of stress relaxation behavior.

Figure 4b shows the expansion, first principal strain of the suture at the sagittal symmetry plane, and Fig. 4c illustrates the first principal stress in the suture at the sagittal symmetry plane after 29 simulated activations of the RTG. Results in Fig. 4 illustrate that internal stress decayed to negligible values approximately 120 seconds after each simulated activation, and the suture elements were able to withstand the applied total displacement numerically without error. Results from the single activation and series of 29 simulated activations gave confidence that the user-defined relaxation material model would be viable for use in the more complex partial-skull simulations.

Of particular note in Fig. 4b is that for each 0.125 mm appliance activation, the step increase in the strain reduces in magnitude for each activation. The decrease in initial strain upon simulated activation is a result of increasing width used in determining strain as opposed to the initial width. Similarly, the magnitude of the peak stress within the suture reduced for each activation step, Fig. 4c, for the same reasons.

### Incorporation of viscoelasticity in partial skull geometry

The partial skull model was simulated in FEA to evaluate how the utilization of various material property assumptions for the MPS/IMS effected the findings for simulated ME treatment. This comparative analysis aimed to highlight any differences in final expansion results (i.e. maxillary displacements) and suture stress-strain response. Upon averaging the first principal nodal stresses across 27 locations, to be described later in this section, it was found that the meshes studied differed by less than 10%. Given the large patient-to-patient variation expected in tissue material properties and the specific research goals of this study, the convergence to less than 10% was deemed sufficient. As such, the 210,880 element mesh was utilized in all simulations. The four simulation cases have been summarized in Table 2 along with completion results of the full 29-activation simulation. Only the case where the MPS and IMS were assigned compliant linear elastic properties, Case 2, failed to run to full completion as a result of excessive element distortion at suture elements which caused the simulation to fail. Simulation cases that ran to completion each have 291 data points spanning the entire simulation. Nodes from the first molar were used in all cases to determine maxillary displacement results.

**Table 2:** Simulation Case Descriptions and Completion Results for the Four MPS/IMS Material Conditions Tested.

Simulation Case	Simulation Description	Completion Status	Failure Reason (If Applicable)
**1**	MPS/IMS: Neglected	Complete	N/A
**2**	MPS/IMS: Compliant Linear Elastic	Incomplete	Element 208790 in MPS/IMS; Failed at 43203 seconds (Between 3^rd^ and 4^th^ Activation)
**3**	MPS/IMS: Viscoelastic	Complete	N/A
**4**	MPS: Viscoelastic IMS: Fused bone properties	Complete	N/A

As highlighted in Table 3, there were negligible differences in displacement between the model where the MPS and IMS were simulated using viscoelastic suture properties (Case 3) and where it was neglected via removal of the symmetry boundary condition on the suture (Case 1). In light of the result that predicted stress within suture tissue decays to negligible values within minutes of activation, it should not be expected to provide increasing resistance to expansion as simulated activations progress. Conversely, displacement results from Case 4 differed significantly when the IMS was assumed fused. Given the added stiffness in the anterior region of the maxilla that would be induced through considering the IMS to be composed of bone, it would then generate larger displacement in the posterior region of the maxilla due to its comparatively lower stiffness. Case 2 resulted in similar displacements to Case 1 and 3 on initial simulated activations, but was unable to predict any results beyond the third activation as a result of simulation failure due to element distortion.

**Table 3:** Average Nodal Displacement of all nodes in the First Molar in the Transverse, Anterior-Posterior, and Vertical Directions for the Four Simulated Cases After 1, 3, 6, and 29 Activations (Coordinate system used is identified in Fig. 3).

Simulation Case	Displacement	Activation Number			
		1	3	6	29
1	X-Component; Transverse Direction (mm)	−0.14	−0.42	−0.84	−4.03
2		−0.14	−0.38	N/A	N/A
3		−0.15	−0.43	−0.84	−4.03
4		−0.17	−0.51	−1.03	−4.92
1	Y-Component; Anterior-Posterior Direction (mm)	0.01	0.03	0.07	0.36
2		0.01	0.03	N/A	N/A
3		0.02	0.04	0.07	0.36
4		0.03	0.08	0.16	0.64
1	Z-Component; Vertical Direction(mm)	−0.02	−0.04	−0.08	−0.29
2		−0.02	−0.04	N/A	N/A
3		−0.02	−0.05	−0.09	−0.29
4		−0.02	−0.07	−0.13	−0.42

**Figure 5:**
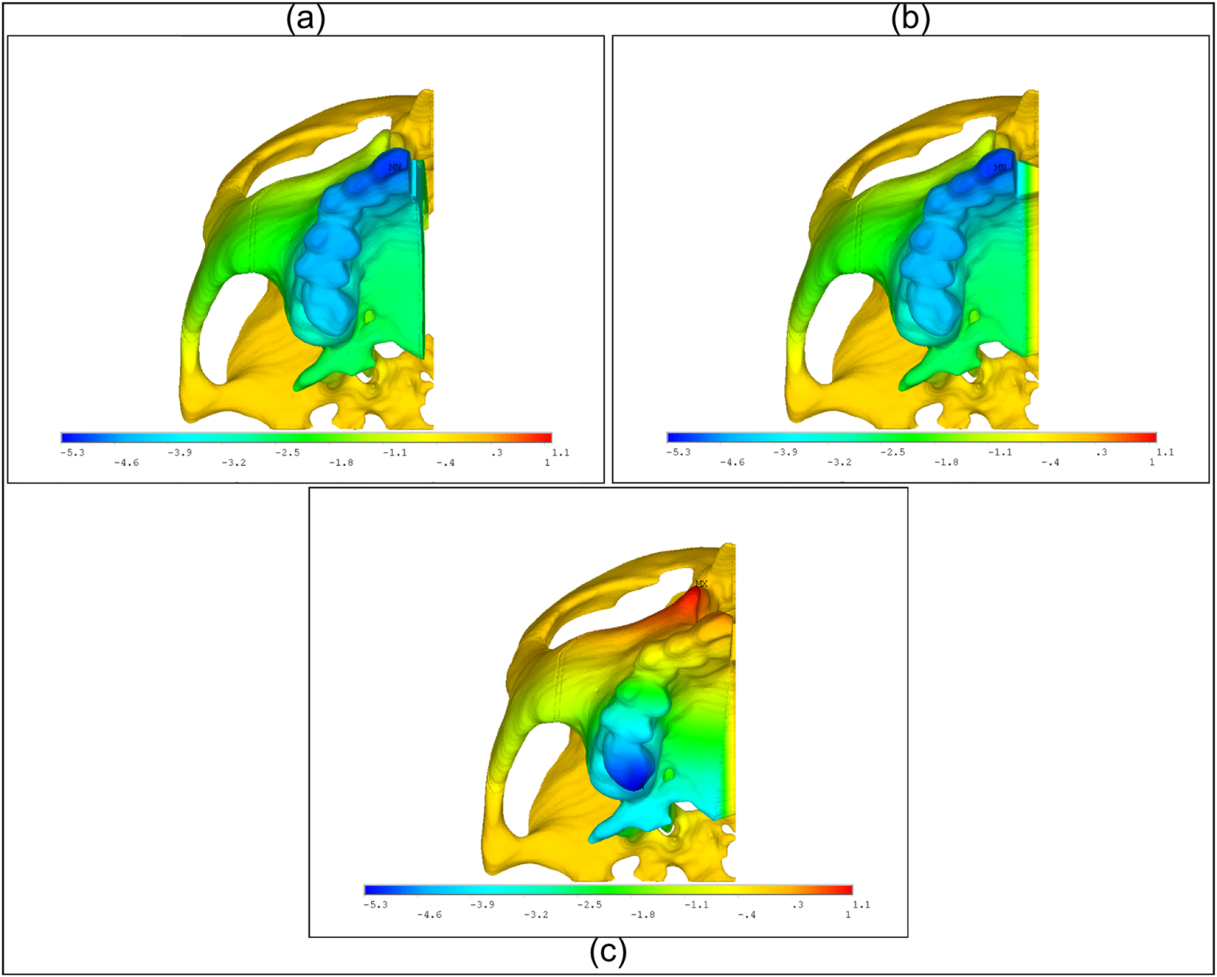
Partial skull displacement fringe patterns showing the x-direction (transverse) displacement of the maxilla for (**a**) neglected MPS/IMS structure, (**b**) viscoelastic MPS/IMS properties, and (**c**) a fused IMS and viscoelastic MPS. By the coordinate system used here, outward lateral displacement. Units are in mm, and the coordinate system used is depicted in Fig. 3.

Considering the maxillary displacements of Cases 1, 3, and 4 further, Fig. 5 illustrates the superior view of the models showing the x-component (transverse) of displacement at the end of the 29^th^ activation. The continuous MPS/IMS simulations where they were both either neglected (Case 1) or assumed viscoelastic (Case 3) show that there is more anterior first molar displacement, while the discontinuous MPS/IMS simulation with a fused IMS and viscoelastic MPS (Case 4) shows that there is more posterior displacement. Work by Cross and McDonald^17^ indicated differing clinical anterior-posterior expansion results from previous work by da Silva *et al*.^18^, for similar ME protocols, regarding whether more expansion was achieved posteriorly or anteriorly. Differences in findings were primarily attributed to inconsistent age groups and expected levels of suture maturation/ossification. It is not suggested here that a fully fused IMS and unfused MPS necessarily represents a common clinical scenario; however, the predicted FEA result in this study that greater posterior expansion may occur over anterior, or vice versa, has been noted through clinical studies in the literature. As such, findings in this study further point to the understanding that careful attention must be paid to assumptions of suture maturity/ossification and interpreting results. That is, FEA of ME treatment may provide a physically representative interpretation of clinical findings provided that appropriate assumptions (e.g. degree and location of ossification) are made about the relevant suture properties that emulate the desired population being considered.

When interpreting FEA displacement/expansion results with regards to clinical relevance, all cases were similar in regards to predicting preliminary displacements. Displacement results showed increasing discrepancies between Cases as simulated activations progressed. If the primary clinical question is to predict the amount of expansion that may be achieved through ME treatment, it is supported by findings from Case 1 and 3 that the MPS/IMS suture structure, if unfused, may be neglected. Given the substantial complexity of incorporating a representative viscoelastic model for the MPS/IMS suture structure, as highlighted in the Methods section, dramatic model simplification could be achieved through removal of the MPS/IMS structure. Caution of course must be exercised around doing so, as the MPS/IMS structure should truly be unfused, or at least within reasonable approximation, in order to neglect it while attaining physically representative results. As noted from Case 4, introduction of a fused IMS certainly changed displacement patterns and results compared to Cases 1 and 3. This again points to making appropriate assumptions in analysis that reflect the desired population being studied and their expected level of suture ossification/maturity.

**Figure 6:**
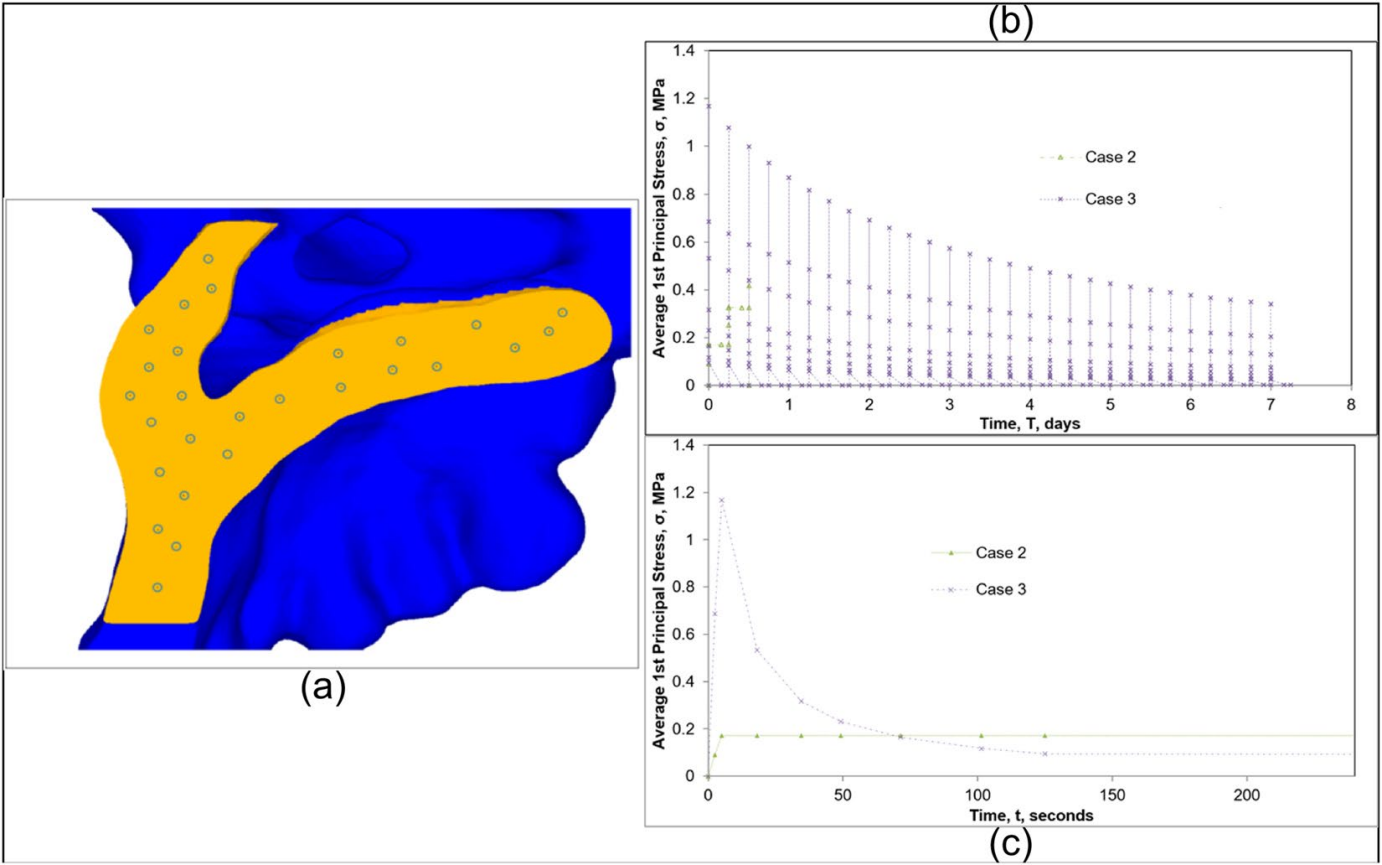
(**a**) Location of the 27 nodes at the sagittal plane of symmetry used to calculate average stress values within the MPS/IMS structure; (**b**) Average first principal stress results in the MPS/IMS structure for entirety of the simulated treatment; (**c**) Average first principal stress in the MPS/IMS structure after a single activation.

To better understand the rates of relaxation in the MPS/IMS structure, 27 nodes were selected within the suture, as illustrated in Fig. 6a. The averaged nodal first principal stress results from the chosen nodes were plotted versus time in Fig. 6b for Case 2 and 3. These results show the relaxation of stresses in the MPS/IMS structure as it goes through subsequent activations using the viscoelastic model compared to the solution assuming compliant linear elastic properties of the MPS and IMS. To isolate a single expansion, Fig. 6c again compares Case 2 and 3 but only for a single activation up to approximately 2 minutes.

In studying the results around stresses developed in the MPS/IMS during simulated ME, it is clear that assuming compliant linear elastic (Case 2) versus viscoelastic (Case 3) suture material properties gave rise to significantly different initial stress profiles in the MPS/IMS structure. Of course, one should not expect to observe relaxation or differential initial stress response for a linear elastic model given that, by definition, there is no time-dependent term in the constitutive relationship; however, the difference between predicted initial stress values within the MPS/IMS structure is clearly illustrated with the viscoelastic approach showing an average initial first principal stress of approximately 1.2 MPa compared to the 0.2 MPa predicted with compliant linear elastic properties. As this result pertains to specific study of ME treatment, if FEA study goals are to understand if suture failure will result in response to a prescribed force or displacement protocol under a known/assumed failure criterion for suture tissue, then an assumption of linear elastic properties will not be sufficient to investigate this question. For instance, consider a case of investigating multiple activations, or larger displacements than the 0.125 mm activation simulated here. Based on evidence from this analysis, one would expect the discrepancy between linear elastic and viscoelastic simulations to increase, and this increase may not be in a linear fashion. Investigating such research questions would necessitate that viscoelastic properties be considered. Results as presented in Fig. 6 do converge after a short period of time to similar values within the 2 minute window presented which could indicate that for a single activation the compliant linear elastic model may predict stress within the suture structure adequately; however, going beyond this would not be appropriate as the effects of stress relaxation are not incorporated and would give rise to misleading findings.

Another topic of interest that necessitates the study of stresses within the MPS/IMS structure is with respect to bone remodeling. It is understood that the application of external mechanical stimuli can induce bone remodeling/growth at suture sites^5^, and is still a topic of significant interest in the literature^19–21^. With respect to ME treatment, it is generally suggested that increased bone remodeling during treatment may lead to improved retention or reduced retention times as a result of increased bone at the suture site preventing relapse. In studying such a phenomenon, it is imperative that the stress-strain response of the suture tissue is appropriately modeled and understood when correlating predicted mechanical FEA results with biological findings. As illustrated here, the stress response of the MPS/IMS structure differed greatly between viscoelastic and compliant linear elastic approaches in terms of initial stress upon application of displacement. If predicting failure of the suture during load application is a goal of the study, especially in instances of rapid displacement application, then it has been shown here that incorporation of the viscoelastic model is necessary to capture the nature of suture response. Conversely, after approximately 2 minutes of simulation time, the viscoelastic and linear elastic models converge to similar values indicating that suture response over time for a single activation could be reasonably predicted using a linear elastic approach. Such an observation has large implications towards studying suture mechanobiology where bone remodeling/growth will take place over time during continual load application. The current study only considered skeletal response to a simulated stepwise increase in displacement (e.g. a Hyrax device), and future work should investigate limitations when ME treatment is performed via a load-controlled device (e.g. spring).

Overall, the individual simulation cases presented in this study have particular instances when their practicality and physical representativeness are appropriate based on the specific research question and population being considered. Removal of the MPS/IMS structure may yield accurate results with respect to understanding displacement/expansion of the maxilla over a simulation of multiple activations, but of course cannot produce any prediction of stresses or strains within the MPS/IMS. Furthermore, removal of the suture may not be physically representative if there is substantial ossification present, as could be the case in an adult patient, for example. Simulation utilizing a linear elastic assumption for suture behavior may provide a reasonable prediction of maxillary displacements and suture stresses or strains for a time period shortly after a single appliance activation. If the initial suture response to activation, namely stress, is of paramount interest (e.g. in the case of studying suture failure), it has been illustrated here that a viscoelastic material model should be considered. Inclusion of suture viscoelasticity does yield physically representative simulation results in terms of studying MPS/IMS stress and strain and maxillary displacement; however, such a high fidelity and complex model also resulted in substantial time for development and solution time. For certain aforementioned research questions it would be necessary to include suture viscoelasticity, but simplifications exist and should be considered where appropriate for expediting solution time.

## Limitations

The element choice (SOLID185) and number of elements for the partial skull analysis were proven to be adequate for the specific research questions investigated in this study, namely the comparison of average nodal first principal stress throughout the midpalatal suture and predicted expansion for varied midpalatal suture material models. Should the research question change to involve a more rigorous evaluation of local material response (e.g. stress or strain at specific locations within the suture or surrounding bone), then it is possible that a different element type or mesh density may be required for converged results. It is also possible that the threshold of convergence may require alteration. This is a critical component of FEA studies and should be considered on a case-by-case basis. Additionally, it is recommended by ANSYS that cubic and prism formulations are used for the SOLID185 element type over the tetrahedral option. While it is not anticipated that the general conclusions of this research would differ with element choice as supported by the convergence of mesh density and nonlinear analysis, specific results may change with nodal configuration (e.g. allowing for simulation of more activations with the linear elastic material model).

In the case of using linear elastic properties for the MPS/IMS structure, Case 2, it was found that all desired activations could not be achieved due to excessive element distortion causing failure. Future work could investigate how increasing the density of the mesh in the MPS/IMS region influences the ability of a linear elastic model for the MPS/IMS to simulate ME treatment at later stages of expansion. While the mesh used in this study resulted in simulation failure early in treatment, it is possible that with additional refinement there may be an increased ability to predict expansion further into simulated treatment. Results from this work do, however, illustrate an inherent potential limitation of using linear elastic elements for the MPS/IMS structure when simulating ME treatment over a series of expansion steps. Additionally, the linear elastic elements do not have the ability to represent the inherent decay in stress over time, and thus would continually superimpose stress results for subsequent activations and likely lead to erroneous stress results in later simulations.

The results and discussion presented in this manuscript must be taken in context as not to mislead the reader. The intent of this research was to incorporate a validated representative viscoelastic model for the MPS/IMS structure in a representative geometric model for simulation of ME treatment, and understand the implications of this compared to neglecting suture material and using linear elastic approaches. Shortcomings and limitations certainly exist with the presented work and must be considered when interpreting results. No bone interdigitation throughout the suture or any bone remodeling throughout treatment was considered, and the MPS/IMS was assumed either fully unfused or fused. Displacements were applied directly to nodes on the maxilla, and not through a simulated appliance which would alter the amount of displacement achieved. The MPS/IMS structure was taken as a uniform symmetric thickness. Finally, only stepwise displacement types of expansion was simulated, and future work should investigate how utilization of other appliances, namely a spring appliance, influences ME treatment simulations. Notwithstanding these limitations, the presented work still provides valuable insight into the inclusion of suture viscoelasticity in simulation of ME treatment and how it influences results.

## Conclusions

The presented study made methodological headway towards understanding predictive FEA models of maxillary expansion treatment. Romanyk *et al*.’s 1-D stress relaxation model of the bulk behavior of the MPS was improved and adapted for use in FEA simulations. This implementation correctly replicated the 1-D model’s stress relaxation curve in 3-D simulations, with a well approximated initial peak stress. Partial skull simulations indicated that maxilla displacement is unaffected by the inclusion of the viscoelastic model. As time progressed after simulated activations, the suture structure trended towards negligible internal stress. This indicated that the viscoelastic nature of the suture is inconsequential in affecting final deformation of the skull within simulations that utilize a direct displacement applied to the maxilla. Conversely, the prediction of initial stress within the MPS/IMS structure upon simulated activation was greatly influenced by inclusion of a viscoelastic model. Stresses between linear elastic and viscoelastic simulations for a single activation proceeded to converge to similar values over time.

## Data Availability

The datasets generated and/or analysed during the current study are available from the corresponding author on reasonable request.

## References

[1] Angell E (1860). Treatment of irregularity of the permanent or adult teeth. Part 1. Dent. Cosm..

[2] Lagravere MO, Carey JP, Heo G, Toogood RW, Major PW (2010). Transverse, vertical, and anteroposterior changes from bone-anchored maxillary expansion vs traditional rapid maxillary expansion: A randomized clinical trial. Am. J. Orthod. Dentofacial Orthop..

[3] Cielo CM, Gungor A (2016). Treatment Options for Pediatric Obstructive Sleep Apnea. Curr. Probl. Pediatr. Adolesc. Health Care.

[4] Pirelli P, Saponara M, Guilleminault C (2004). Rapid maxillary expansion in children with obstructive sleep apnea syndrome. Sleep..

[5] Herring SW (2008). Mechanical influences on suture development and patency. Front. Oral. Biol..

[6] Tanaka E, Miyawaki Y, del Poloz R, Tanne K (2000). Changes in the biomechanical properties of the rat interparietal suture incident to continuous tensile force application. Arch. Oral Biol..

[7] Romanyk DL (2013). Role of the midpalatal suture in FEA simulations of maxillary expansion treatment for adolescents: A review. Int. Orthod..

[8] Lee SC (2014). Effect of bone-borne rapid maxillary expanders with and without surgical assistance on the craniofacial structures using finite element analysis. Am. J. Orthod. Dentofacial Orthop..

[9] Serpe LCT, de Las Casas EB, Toyofuku ACMM, Gonzalez-Torres LA (2015). A bilinear elastic constitutive model applied for midpalatal suture behavior during rapid maxillary expansion. Res. Biomed. Eng..

[10] Trojan LC, Gonzalez-Torres LA, Melo ACM, de Las Casas EB (2017). Stresses and strains analysis using different palatal expander appliances in upper jaw and midpalatal suture. Artif. Organs.

[11] Provatidis C, Georgiopoulos B, Kotinas A, MacDonald JP (2006). *In vitro* validated finite element method model for a human skull and related craniofacial effects during rapid maxillary expansion. Proc. Inst. Mech. Eng. H..

[12] Provatidis C, Georgiopoulos B, Kotinas A, MacDonald JP (2007). On the FEM modeling of craniofacial changes during rapid maxillary expansion. Med. Eng. Phys..

[13] Romanyk DL (2016). Viscoelastic response of the midpalatal suture during maxillary expansion treatment. Orthod. Craniofac. Res..

[14] Romanyk DL (2013). Towards a viscoelastic model for the unfused midpalatal suture: Development and validation using the midsagittal suture in New Zealand white Rabbits. J. Biomech..

[15] Fung, Y. C. *Biomechanics: mechanical properties of living tissues* 2nd ed (Springer-Verlag, 1993).

[16] Radhakrishnan P, Mao JJ (2004). Nanomechanical properties of facial sutures and suture mineralization front. J. Dent. Res..

[17] Cross DL, McDonald JP (2000). Effect of rapid maxillary expansion on skeletal, dental, and nasal structures: a postero-antrior cephalometric study. Eur. J. Orthod..

[18] da Silva OG, do Prado Montes LA, Torelly LF (1995). Rapid maxillary expansion in the deciduous and mixed dentition evaluate through posteroanterior cephalometric analysis. Am. J. Orthod. Dentofacial Orthop..

[19] Hou B, Fukai N, Olsen BR (2007). Mechanical force-induced midpalatal suture remodeling in mice. Bone.

[20] Liu SS-Y, Opperman LA, Kyung H-M, Buschang PH (2011). Is there an optimal force level for sutural expansion. Am. J. Orthod. Dentofacial Orthop..

[21] Wu B-H (2017). Stretch forces guides finger-like pattern of bone formation in suture. PLoS One.

